# Immunobiology of Carbohydrates: Implications for Novel Vaccine and Adjuvant Design Against Infectious Diseases

**DOI:** 10.3389/fcimb.2021.808005

**Published:** 2022-01-18

**Authors:** Giuseppe Stefanetti, Francesco Borriello, Barbara Richichi, Ivan Zanoni, Luigi Lay

**Affiliations:** ^1^ Department of Immunology, Blavatnik Institute, Harvard Medical School, Boston, MA, United States; ^2^ Division of Immunology, Harvard Medical School and Boston Children’s Hospital, Boston, MA, United States; ^3^ Department of Chemistry “Ugo Schiff”, University of Florence, Florence, Italy; ^4^ Division of Immunology, Division of Gastroenterology, Harvard Medical School and Boston Children’s Hospital, Boston, MA, United States; ^5^ Department of Chemistry, University of Milan, Milan, Italy

**Keywords:** vaccines, carbohydrates, adjuvants, carbohydrate-based adjuvants, carbohydrate-based vaccines, glycoconjugates, adaptive immunity, innate immunity

## Abstract

Carbohydrates are ubiquitous molecules expressed on the surface of nearly all living cells, and their interaction with carbohydrate-binding proteins is critical to many immunobiological processes. Carbohydrates are utilized as antigens in many licensed vaccines against bacterial pathogens. More recently, they have also been considered as adjuvants. Interestingly, unlike other types of vaccines, adjuvants have improved immune response to carbohydrate-based vaccine in humans only in a few cases. Furthermore, despite the discovery of many new adjuvants in the last years, aluminum salts, when needed, remain the only authorized adjuvant for carbohydrate-based vaccines. In this review, we highlight historical and recent advances on the use of glycans either as vaccine antigens or adjuvants, and we review the use of currently available adjuvants to improve the efficacy of carbohydrate-based vaccines. A better understanding of the mechanism of carbohydrate interaction with innate and adaptive immune cells will benefit the design of a new generation of glycan-based vaccines and of immunomodulators to fight both longstanding and emerging diseases.

## Introduction

### The Glycocalyx and the Immunomodulatory Role of Carbohydrates

Carbohydrates are the most abundant biomolecules on earth and are an essential part of all living organisms (Seeberger and Rademacher, 2014). Carbohydrates are an integral part of cell biology and are exposed on the cell surfaces of all organisms, mainly as components of complex conjugates, such as glycoproteins and proteoglycans, cell surface glycolipids and nucleic acids. The set of cell surface glycoforms constitutes the *glycocalyx*, a key player in the biology of both eukaryotic and prokaryotic cells, involved in multiple vital cellular processes. The glycocalyx protects the cell from ionic and mechanical stress, preserving the integrity of the membrane and acting as a barrier from invading microorganisms. Furthermore, the glycocalyx plays an essential role in every cellular communication and recognition event and it is critical in determining innate and adaptive immune responses to pathogens and in controlling the growth and spread of cancer (Rabinovich et al., 2012; Mahla et al., 2013; Varki, 2017).

In bacteria, the glycocalyx, among other glycan-related structures, comprehends: i) the lipopolysaccharide (LPS) of the outer membrane of Gram-negative species; ii) the peptidoglycan layer of Gram-positive cells; or iii) the polysaccharide coat (capsular polysaccharides, CPS) of encapsulated bacteria, a large group of highly infectious microorganisms that can be either Gram-positive or Gram-negative. Surface polysaccharides are critical for many purposes: they can trigger bacterial adhesion and host cells infection; they exert a protective function against the host’s immune defense, for example interfering with innate immunity by preventing the activation of the alternative complement pathway or by mimicking the host self-antigens; they provide a hydrophilic character to microorganisms that protect them from drying out (Taylor and Roberts, 2005).

Many bacterial carbohydrates are also target of protective antibodies and have been shown to be antigens of interest in carbohydrate-based vaccines development for nearly a century, in particular against encapsulated microorganisms (Jones, 2005; Costantino et al., 2011). In the late 1980s, the first glycoconjugate vaccines, in which the saccharide antigen is covalently linked to a carrier protein, were licensed to overcome the problem of poor immunogenicity of polysaccharide vaccines in young children (Jones, 2005; Costantino et al., 2011; Richichi et al., 2021). Until now, polysaccharide and glycoconjugate vaccines are among the safest and most successful vaccines ever developed.

Microbial carbohydrates (and carbohydrate-containing structures) are also capable of stimulating the innate arm of the immune response ***via*** multiple mechanisms, including interaction with specific pattern recognition receptors (PRRs) on the surface of antigen-presenting cells (APCs) (O’Hagan et al., 2020). By stimulating innate responses, glycans can act as immunomodulators and promote the development of antigen-specific adaptive immunity and have shown adjuvant activity in many pre-clinical and clinical studies (O’Hagan and Fox, 2015).

Based on these premises, our review aims at highlighting how the latest advances in understanding carbohydrates immunobiology can guide the design and development of better vaccines and adjuvants, with a focus on antibacterial glycoconjugate vaccines. We will provide an updated overview of the latest technologies available for the design of carbohydrate-based vaccines, including approaches aimed at an efficient and controlled modulation of immunity. In contrast to other vaccination platforms, many carbohydrate-based antibacterial vaccines do not contain adjuvants to enhance the immune response. We will investigate this interesting observation by describing new progresses on the generation of adjuvants for glycoconjugate vaccines and by focusing on the immunomodulatory properties of glycans that hold promise as vaccine adjuvants. In a nutshell, we will focus on how the dual role of carbohydrates as antigens, recognized by the adaptive immune response, and adjuvants, targeting the innate immune response, could lead to a new generation of vaccines with improved efficacy.

### Carbohydrate-Based Vaccines and the Need for T Cell Help

The story of carbohydrate-based vaccines dates back to 1923, with the discovery of Avery and Heidelberger that CPS from *Streptococcus pneumoniae* are immunoreactive (Heidelberger and Avery, 1923; Heidelberger and Avery, 1924). CPS-specific neutralizing antibodies were also found to mediate protection against pneumococcal infections (Finland and Sutliff, 1932), and the first CPS-based vaccine, targeting four *S. pneumoniae* serotypes, became available in 1945 (MacLeod et al., 1945). However, the advent of chemotherapeutics and antibiotics in the following years dampened the enthusiasm towards the vaccination practice, based on the general belief that antibiotics could represent the panacea for all infectious diseases. It was only a few years later, in the 1960s, that the emergence and the constant increase of multidrug resistance phenomenon generated concerns on the use of antibiotic to fight infections and led to a renewed interest in preventive strategies, giving new impetus to the development of carbohydrate-based vaccines.

Since then, extensive literature has highlighted the role of carbohydrate-specific antibodies in preventing microbial infections and led to the approval of CPS-based monovalent and multivalent vaccines against *S. pneumoniae*, *Neisseria meningitidis*, *Haemophilus influenzae* type b (Hib), and *Salmonella* Typhi (**Table 1**). The first anti-meningococcal polysaccharide-based vaccine, MPSV4, was introduced in 1978 (Prevention and Control of Meningococcal Disease). PneumoVax (Merck and Co.), composed of unconjugated CPS isolated from 14 serotypes of *S. pneumoniae*, was licensed and marketed in the United States in 1977, while the current version, introduced in 1983, includes 23 of the approximately 90 known serotypes (Robbins et al., 1983). In the following decades other CPS-based vaccines were launched, targeting Hib (1985, produced by Praxis Biologics), a major cause of bacterial respiratory tract infections that can lead to severe diseases such as pneumonia, sepsis, and meningitis, and *S.* Typhi (produced in France by Pasteur Mérieux and introduced in the USA in 1994 by Connaught Laboratories), a leading cause of typhoid fever (Plotkin and Plotkin, 2011).

**Table 1:** Carbohydrate-based vaccines approved by the FDA.

Commercial name	Manufacturer	Antigen	Adjuvant
**Glycoconjugate vaccines with adjuvants**			
**Liquid PedvazHIB**	Merck Sharp & Dohme	*Haemophilus influenzae* type b; CPS (polyribosyl-ribitol-phosphate)	Amorphous aluminium hydroxyphophate sulfate
**Pentacel**	Sanofi Pasteur	*Haemophilus influenzae* type b; CPS (polyribosyl-ribitol-phosphate)	Aluminium phosphate
**VAXELIS**	MCM Vaccine	*Haemophilus influenzae* type b; CPS (polyribosyl-ribitol-phosphate)	Aluminium salts
**Prevnar 13**	Wyeth Pharmaceuticals	*Streptococcus pneumoniae* serotypes 1, 3, 4, 5, 6A, 6B, 7F, 9V, 14, 18C, 19A, 19F, and 23F; CPS	Aluminium phosphate
**Prevnar 20**	Wyeth Pharmaceuticals	*Streptococcus pneumoniae* serotypes 1, 3, 4, 5, 6A, 6B, 7F, 8, 9V, 10A, 11A, 12F, 14, 15B, 18C, 19A, 19F, 22F, 23F and 33F; CPS	Aluminium phosphate
**VAXNEUVANCE**	Merck Sharp & Dohme	*Streptococcus pneumoniae* serotypes 1, 3, 4, 5, 6A, 6B, 7F, 9V, 14, 18C, 19A, 19F, 22F, 23F and 33F; CPS	Aluminium phosphate
**Glycoconjugate vaccines without adjuvants**			
**HIBERIX**	GlaxoSmithKline Biologicals	*Haemophilus influenzae* type b; CPS (polyribosyl-ribitol-phosphate)	—–
**ActHIB**	Sanofi Pasteur	*Haemophilus influenzae* type b; CPS (polyribosyl-ribitol-phosphate)	—–
**Menactra**	Sanofi Pasteur	*Neisseria meningitidis serogroups A, C, Y and* W-135; CPS	——
**MENVEO**	GlaxoSmithKline Biologicals SA	*Neisseria meningitidis* serogroups A, C, Y and W-135; CPS	—–
**MenQuadfi**	Sanofi Pasteur	*Neisseria meningitidis* serogroup W (MenQuadfi); CPS	—–
**Typhim Vi**	Sanofi Pasteur	*Salmonella enterica* serovar Typhi; cell surface Vi polysaccharide	—–
**Polysaccharide vaccines (all without adjuvants)**			
**Menomune-A/C/Y/W-135**	Sanofi Pasteur	*Neisseria meningitidis* serogroups A, C, Y and W-135; CPS	—–
**Typhim Vi**	Sanofi Pasteur	*Salmonella enterica* serovar Typhi; cell surface Vi polysaccharide	—–
**PNEUMOVAX 23**	Merck & Co.	*Streptococcus pneumoniae* serotypes 1, 2, 3, 4, 5, 6B, 7F, 8, 9N, 9V, 10A, 11A, 12F, 14, 15B, 17F, 18C, 19F, 19A, 20, 22F, 23F, and 33F; CPS	—–

This table was adopted from the complete list of ‘Vaccines Licensed for Use in the United States’ provided by the US Food and Drug Administration website (https://www.fda.gov/). CPS: capsular polysaccharide.

Content current as of: November 1, 2021.

However, it was soon discovered that polysaccharide vaccines are not effective in children under 2 years old, and only poorly immunogenic in young, old and immunocompromised subjects. Even in adults, the induced humoral response is short-lasting and fails to generate conventional B cell-mediated immunological memory (Segal and Pollard, 2004; González-Fernández et al., 2008). The limited clinical efficacy of polysaccharide-based vaccines is largely attributed to the T cell-independent immune response they induce (Mond et al., 1995; Rappuoli, 2018).

The original discovery in the 30s that the polysaccharide immunogenicity can be strongly enhanced by their conjugation (i.e. covalent linking) to an immunogenic carrier protein (Avery and Goebel, 1929; Avery and Goebel, 1931) was revisited in the 80s, and led to the development of glycoconjugate vaccines, a key breakthrough in the field of vaccinology. Glycoconjugate antigens can elicit a T cell-dependent response, resulting in the production of antibodies of increased affinity and in the generation of carbohydrate-specific memory B cells (MBCs). As a result, glycoconjugate vaccines are protective in young children (less than 2 years old) and, overall, more effective than polysaccharide vaccines (Ada and Isaacs, 2003; Pollard et al., 2009). For about 35 years, glycoconjugate vaccines have been used to successfully protect infants, adolescent, and adults from a variety of bacterial diseases (**Table 1**). The drastic reduction of *S. pneumoniae*, Hib and *N. meningitidis* infections in Western countries demonstrates the power of this intervention. Glycoconjugate vaccines have also been a market success. For example, the 13-valent pneumococcal vaccine PCV13 (Prevnar 13, Pfizer) achieved 6 billion USD sales in 2019, making it Pfizer’s best-selling solo medicine (FiercePharma, 2021).

The first glycoconjugate vaccine targeted the Gram-negative bacterium Hib and was authorized in several formulations between 1987 and 1990 (Schneerson et al., 1980; Eskola et al., 1987; Black et al., 1991). Likewise, mono- and polyvalent meningococcal conjugate vaccines became available between 1999 and 2005 (Bröker et al., 2016), while the first pneumococcal glycoconjugate vaccine containing seven serotypes (PCV7) was introduced in 2000 in the United States (Yildirim et al., 2015). In the last years, two *S.* typhi conjugate vaccines were also licensed (Micoli et al., 2011; Mohan et al., 2015). Although existing conjugate vaccines have proven very effective, variations in the geographic distribution of serotypes as well as the serotype replacement phenomenon may impair their ability to control disease burden. As a result, WHO strongly recommends careful monitoring of vaccines’ effectiveness, and vaccine manufactures are constantly evaluating new formulations to improve and expand serotype coverage. For example, a 15-valent (Stacey et al., 2019) and a 20-valent (Hurley et al., 2021) pneumococcal conjugate vaccines have been approved very recently (2021) by the Food and Drug Administration (FDA) with the trade names VAXNEUVANCE and PREVNAR 20, respectively.

Glycoconjugate vaccines were developed to overcome the limited protection provided by polysaccharide vaccines. Since then, new technologies have been proposed both to improve their immunogenicity and to reduce the variability of their molecular structure, which complicates the analytical characterization and can cause lot-to-lot variation in the immune response generated by the vaccine. In addition, the use of the multivalent design has been proposed which allows the co-tagging of adjuvants or other molecules of interest. In the next chapters we will examine the state of the art of new approaches utilized to generate carbohydrate-based vaccines.

### Carbohydrate-Based Vaccines and Adjuvant Research

The immune response to vaccine antigens can be enhanced by using immunostimulatory components, known as adjuvants. Named after the Latin word *adiuvare* (to aid or help), adjuvants are substances or molecules able to accelerate, prolong, or amplify the specific response to antigens. Given that nowadays many vaccines are developed from purified components of pathogens, adjuvants are required to enhance the immune response. The most widely used adjuvants are aluminum salts which were first used by the immunologist, Alexander T. Glenny, in 1926 at the Wellcome Physiological Research Laboratory in London (Glenny et al., 1926). Besides aluminum salts, only few other adjuvants have been approved for use in human vaccines. However, recent technological advances in the field led to licensing of new adjuvants for several products, including the protein-based malaria, influenza, human papilloma virus (HPV) and varicella zoster virus vaccines (O’Hagan et al., 2020). Furthermore, there is an increased understanding of the immunomodulatory actions of adjuvants and of the molecular mechanisms that drive immune cell activation (Schijns et al., 2020; Immunopotentiators in Modern Vaccines - 2nd Edition., 2021). In particular, adjuvants design has been significantly inspired by the processes that regulate antigen delivery/presentation. Indeed, to date, the term adjuvants include both delivery systems able to target antigens to specific cells and structurally different ligands that target the PRRs expressed by innate immune cells including Toll-like receptors (TLRs), nucleotide-binding oligomerization domain (NOD)-like receptors (NLRs), retinoic acid-inducible gene I (RIG-I)-like receptors (RLRs), and C-type lectin receptors (CLRs). These ligands activate the immune response of innate immune cells, such as dendritic cells (DCs). PRRs evolved to recognize pathogen-associated molecular patterns (PAMPs), and/or danger associated molecular patterns (DAMPs), thus triggering DCs maturation, the release of pro-inflammatory mediators, and migration to secondary lymphoid organs (Anderluh et al., 2021). In addition to adjuvants that target the PRRs, the use of cytokines and/or chemokines has been implemented to avoid the unwanted local reactions driven by PRR stimulation, and the use of squalene-based emulsions have also been proposed and/or licensed (Degen and Schijns, 2017; Kommareddy et al., 2017).

Aluminum salts are the only adjuvants licensed for carbohydrate-based vaccines, but the benefit of their use is not always present. Indeed, the inclusion of aluminum salts does not enhance the response to polysaccharide vaccines and not all the glycoconjugate vaccines contains an adjuvant (**Table 1**). In the light of the above, part of the review will be dedicated at addressing two separate but interdependent questions: 1) what the most recent advancement in the development of adjuvants for glycoconjugate vaccines against infectious diseases are and 2) how the use of carbohydrates-based adjuvant vaccines by the targeting of PRRs can provide an efficient delivery of the bacterial antigenic epitopes and, in turn an effective activation of the innate immune cell compartments.

## Immune Responses to Carbohydrate-Based Vaccines

### Polysaccharide Vaccines

**Figure 1:**
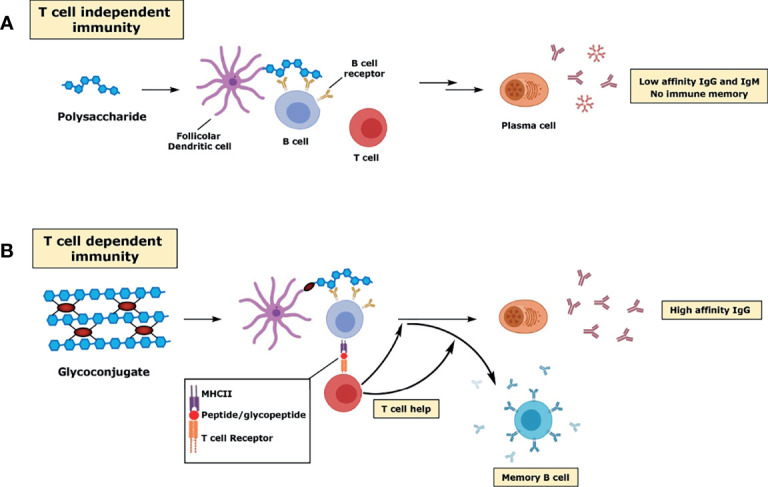
Schematic representation of the immune response to polysaccharides **(A)** and glycoconjugates **(B)**.

Polysaccharide vaccines are licensed and used in many countries, but their use has some limitations and is age specific. Remarkably, polysaccharides do not induce immune responses in infants under the age of two and therefore cannot be used as vaccines in this population. Even in adults, vaccination with polysaccharide vaccines is suboptimal as it does not induce immunological memory, avidity maturation and isotype switching. Most of the antibodies produced are IgM and IgG2, which are only poorly complement activators, therefore less able to fight pathogens (Musher et al., 1990; Lortan et al., 1993). Furthermore, although necessary to maintain a sufficient level of antibodies, repeat vaccination does not lead to increased antibody titers, but instead triggers hyporesponsiveness, with the polysaccharide-induced apoptosis of MBCs and consequent reduced immune response to subsequent immunizations (Richmond et al., 2000). Polysaccharides are T cell-independent type 2 (TI-2) immunogens, based on the mechanism by which they activate B cells (**Figure 1A**). TI-2 antigens consist of highly repetitive epitopes, such as the polymer backbone of polysaccharides. Differently from T cell-independent type 1 (TI-1) immunogens (e.g. LPS or bacterial DNA), TI-2 immunogens do not have an intrinsic B-cell activating activity, but instead rely on cross-linking of approximately 15-20 B cell receptors (BCR), which initiates signaling by prompting a protein phosphorylation cascade leading to an increase in free intracellular calcium (Snapper and Mond, 1996). Both DCs and macrophages have been shown to provide important co-stimulatory signals for initial B cell stimulation (Balázs et al., 2002; MacLennan and Vinuesa, 2002; Craxton et al., 2003). After activation, B cells mature into plasma cells and secrete antibodies, without the development of MBCs (**Figure 1A**). The mechanism of B cell activation, based on the crosslink of multiple BCRs, explains why the immunogenicity of these vaccines is size-dependent, with only the high molecular weight antigens being able to induce an effective immune response. T cell–independent immune responses generally involve B-1 cells, a subpopulation of non-conventional B cells that replicates autonomously, and marginal zone (MZ) B cells, non-circulating mature B cells that segregate in the MZ of the spleen or in other lymphoid tissues.

Interestingly, the meningococcus A polysaccharide vaccine appears to be an exception to the above-mentioned limitations. This vaccine induces formation of MBCs and allows a good immune response even in infants (Gold et al., 1977). The reasons for this unique behavior are currently unknown (Rappuoli, 2018). In addition, a specific class of glycans, zwitterionic polysaccharides (ZPS), also induces T cell responses. The proposed mechanism of T cell activation relies on the presence of both positive (e.g. free amine) and negative (e.g. phosphate or carboxylate) charges that allow the processed antigen to be presented by major histocompatibility complex class II molecules (MHCII) by APCs (e.g. DCs, B cells and macrophages) and to be recognized by T cell receptors (TCRs) (Tzianabos et al., 1993; Kalka-Moll et al., 2002; Cobb et al., 2004).

### Glycoconjugate Vaccines

As discussed above, a major problem with polysaccharide vaccines is the poor immunogenicity induced in children under 2 years of age. Proteins and peptides induce T cell-dependent (TD) responses, as they can stimulate helper T lymphocytes. Unlike TI antigens, TD antigens are immunogenic even in early childhood. The immune response elicited against TD antigens can be both boosted by multiple vaccinations and enhanced by adjuvants. Finally, B cell activation upon TD antigens encounter is characterized by the development of plasma cells inducing high affinity antibodies and MBCs. Conjugation of proteins or peptides to carbohydrates can therefore be considered as an “artificial” way to provide T cell epitopes (i.e. peptides) which are needed in germinal centers (GCs), sites within lymph nodes and the spleen where the B response develops, for affinity maturation of polysaccharide-specific B cells. Since the introduction of the hapten-carrier concept in 1929 (Avery and Goebel, 1929; Goebel and Avery, 1929; Avery and Goebel, 1931), the response to conjugate vaccines has been investigated and reviewed in several studies (Jones, 2005; Costantino et al., 2011). The conjugate vaccine is taken up by DCs at the site of immunization and, within a few days, is transported to the lymph nodes where the presence of a TD antigen (the carrier protein) prompts the development of GCs. Within the GC, the processed glycoconjugate binds the surface immunoglobulin (sIg) of B cell specific for the saccharide hapten, and over a series of processes mature B cells proliferate, differentiate, and mutate their antibody genes through somatic hypermutation which results in the development of higher affinity antibodies as well as class switching (mainly from IgM to IgG) (**Figure 1B**). The dynamic changes in GCs occur in spatially distinct regions of the GC, the light and the dark zone, and involve specific class of follicular immune cells (e.g. T follicular helper (TFH) cells and follicular dendritic cells (FDCs)). As a result, selected B cells exits the GC to become MBCs and antibody-secreting plasma cells (Rappuoli, 2018) (**Figure 1B**). The different mechanisms of immune response towards polysaccharide and glycoconjugate antigens have also practical consequences in vaccine design. Since crosslinking of surface immunoglobulin molecules on B cells is not required, glycoconjugate vaccines can also be produced from small saccharide chains.

The mechanism of glycoconjugate vaccine processing by APCs and presentation to T cells has also been investigated. Following internalization of the conjugate vaccines by APCs endosomes, the polysaccharide part is depolymerized into smaller carbohydrates by oxidative agents such as reactive oxygen species (ROS) and reactive nitrogen species (RNS) (Avci et al., 2011; Sun et al., 2019). The protein portion is instead processed by acidic proteases into peptides. After this initial processing, small peptides are then presented to T lymphocytes in association with MHCII molecules. T cells provide appropriate signals both through direct interactions of cell surface proteins and *via* cytokine signaling processes, to induce maturation of the B cells into either antibody secreting plasma cells or MBCs. The role of B-T cell co-signaling in the context of conjugate vaccines presentation is still yet not fully understood, but B7-CD28 and CD40-CD40L interactions have been shown to be critical for immune responses *in vivo (* Guttormsen et al., 1999).

Importantly, recent studies suggest that glycopeptide fragments resulting from the glycoconjugate processing inside the B cell can also bind to MHCII molecules whereas the hydrophilic carbohydrate portion is exposed to the TCR, where it can interact with carbohydrate-specific CD4+ T cells (T carb) (Avci et al., 2011; Avci et al., 2012) (**Figure 1B**). More recently, it has been shown that a similar mechanism applies to conjugates prepared from other CPS (*S*. Typhi Vi CPS, Group B Streptococcus type Ib, Hib), except that group C *N. meningitidis* CPS, where only peptides generated from the carrier protein were critical for helper T cell recognition. This study showed that different mechanisms of presentation, based on the structure of the carbohydrate, are operative in response to different glycoconjugate vaccines (Sun et al., 2019). Carbohydrate-specific T cell–mediated humoral responses have been also shown with glycoconjugates of type 3 *S. pneumoniae* CPS (Middleton et al., 2017).

## Carbohydrate-Based Vaccines: Challenges and New Opportunities

The development of carbohydrate-based vaccines relies on the production by the immune system of antibodies that mediate protection against the specific targeted pathogen. As seen before, the humoral response generated by polysaccharide vaccines is T-cell independent and hence the need to generate glycoconjugates to improve overall the immunogenicity and to ensure protective efficacy in young children. However, despite the huge impact that conjugate vaccines have had on global health over the past 30 years, there are still some limitations to the use of this vaccination approach. Although conjugate vaccines have been very effective overall, some immunogenicity issues persist for certain groups at high risks, such as the elderly or immunocompromised individuals, where immunogenicity has been relatively poor (Avci and Kasper, 2010). Furthermore, most glycoconjugate vaccines require few booster doses to achieve full protection and are unable to rapidly induce protective antibody titers (Buonsanti et al., 2016). Another complication is due to variations in global serotype distributions and serotype replacement events, such as for pneumococcal and meningococcal diseases, which require constant monitoring of the vaccine serotype coverage (Herva et al., 1980; Geno et al., 2015; Ji et al., 2017) and the inclusion of new serogroups in already licensed vaccines (Stacey et al., 2019; Hurley et al., 2021). Additionally, the use of alternative protein carriers to improve vaccine immunogenicity has been recommended, particularly when dealing with multivalent formulations. Indeed, most licensed conjugate vaccines use the same set of carrier proteins, such as tetanus toxoid (TT), diphtheria toxoid (DT) and CRM_197_, and preexisting immunity to the protein (‘carrier epitope suppression’) has been associated in certain circumstances with reduced immunogenicity against the polysaccharide hapten (Dagan et al., 2010). Moreover, from an economic and sustainability point of view, the high-cost technology and the expertise required to develop effective conjugate vaccines make their use in developing countries problematic.

### Improving the Immunogenicity of Glycoconjugate Vaccines

**Figure 2:**
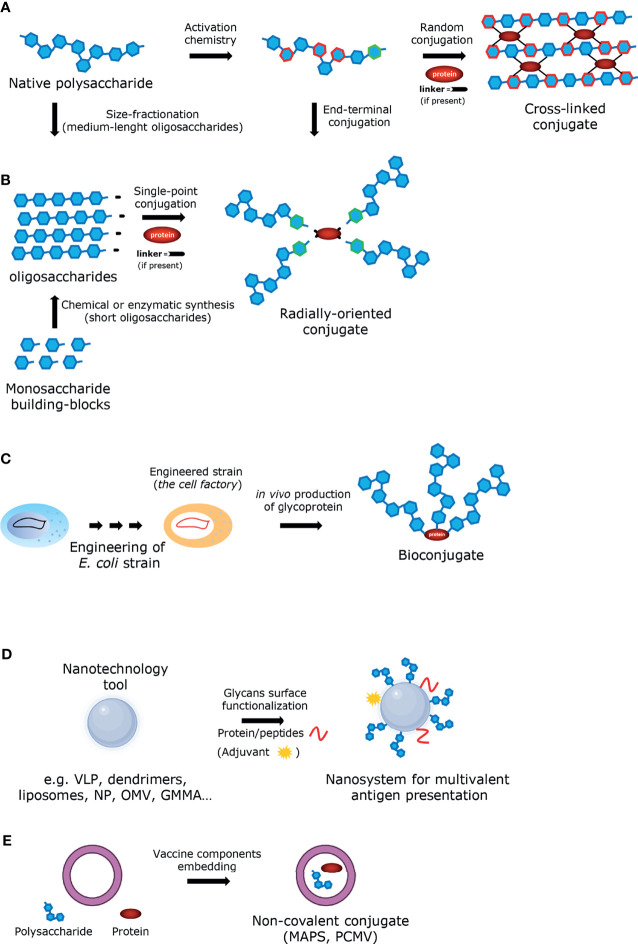
Graphic illustration of the approaches employed for the construction of glycoconjugate vaccines **(A)** Glycoconjugate obtained by random conjugation of native polysaccharide. **(B)** Glycoconjugate obtained by end-terminal conjugation of oligosaccharides (produced either by size-fractionation of polysaccharide or by chemical/enzymatic synthesis). **(C)** Bioconjugate obtained *in vivo* from engineered *E. coli* strain. **(D)** Glycoconjugate obtained from nanotechnology tool. **(E)** Non-covalent conjugate obtained by entrapment of the vaccine components.

Many variables affect the immunogenicity of conjugate vaccines including the saccharide structure and the presence of non-saccharide substituent (e.g. O-acetyl groups), the saccharide and the glycoconjugate size, the carrier protein, the saccharide to protein ratio (Jones, 2005; Costantino et al., 2011; Khatun et al., 2017). Also, the conjugation chemistry (Richichi et al., 2021), including the presence of the linker (Costantino et al., 2011) and the attachment point on the carrier protein (Stefanetti et al., 2015), may play an important role. A better understanding of these parameters can lead to improved rational design of glycoconjugate vaccines, but it is not straightforward because: 1) most glycoconjugates are prepared by following classical random conjugation methods which result in heterogeneous mixtures of high molecular weight, cross-linked and structurally-undefined molecules (Berti and Adamo, 2018) (**Figure 2A**); 2) the immune response is highly antigen dependent, and so contrasting findings can be obtained working with different sugar haptens; 3) most of the studies performed so far have compared the immunogenicity of vaccines differing for several parameters at the same time, thus making it difficult to assign the relative importance of the single variable to immunogenicity; 4) many parameters (i.e. saccharide chain length, saccharide to protein ratio and conjugation chemistry) are strongly interconnected. In this regard, glycoconjugates obtained by site-selective modification of sugar and protein represent promising candidates for a new generation of innovative and effective carbohydrate-based vaccines (**Figure 2B**), offering not only advantages in terms of consistency of production and analytical characterization, but also allowing a better investigation of the structure-immunological activity space (Rabuka, 2010; Nilo et al., 2014; Stefanetti et al., 2015; Hu et al., 2016). The use of bacterial oligosaccharides obtained by organic synthesis allows a further molecular definition of the sugar antigen and guarantees the absence of any microbial contaminants, and it has been seen with increased interest in the last years, also thanks to the development of new technologies such as solid-phase automated synthesis, HPLC-assisted oligosaccharide assembly and enzymatic approaches that have simplified their preparation. The application of these technologies have increased the number of carbohydrate antigens available for the development of glycoconjugate vaccines (Verez-Bencomo et al., 2004; van der Put et al., 2016; Baek et al., 2018). However, the synthetic route remains challenging for some applications mainly due to the difficulty associated with the synthesis of certain structures and to the size-dependent immunogenicity of certain antigens to dimensions difficult to be obtained with “bottom-up” methodologies. The recently described strategies for site-selective protein conjugation may lead to further improvements. Targeting a specific set of amino acids over others present in the protein using fully synthetic saccharide antigens is a very promising approach to provide a new generation of innovative carbohydrate-based vaccines with homogeneous and well-defined structures (Hu et al., 2016; Berti and Adamo, 2018). An alternative source of well-defined glycoconjugate vaccines that has emerged in recent years involves the use of glycoprotein bioengineering (Wacker et al., 2002) (**Figure 2C**). This technology is based on the *N*-linked glycosylation system from *Campylobacter jejuni*, that can be functionally expressed in *Escherichia coli*. The oligosaccharide is assembled on the lipid carrier undecaprenyl-pyrophosphate (Und-PP), to be eventually transferred by the oligosaccharyltransferase (OTase) PglB to the acceptor proteins. By using this approach, both the saccharide antigen and the carrier protein are biosynthesized and coupled in *E. coli* cells (Wacker et al., 2002; Terra et al., 2012). The protein-glycan coupling technology (PGCT) has led to the development of glycoconjugate vaccine candidates against *S. dysenteriae* O1, *S. flexneri* 2a (Hatz et al., 2015), and extraintestinal pathogenic *E. coli (* Huttner et al., 2017), currently being evaluated in clinical trials. The approach can also be adapted to Gram-positive CPS, as recently demonstrated with *S. pneumoniae (* Reglinski et al., 2018). The availability of additional OTase, such as PglL (Faridmoayer et al., 2008) and PglS (Harding et al., 2019), has expanded the tools available for protein glycoengineering. The advantages of this methodology include the possibility of avoiding the manipulation of pathogens, the production of structurally-defined glycoconjugates and an overall simplified vaccine development, compared to traditional methods, with simpler product characterization and reduced production costs. Nevertheless, this methodology has some limitations. This approach has been shown to be suitable for the Wzx/Wzy and ABC polysaccharide biosynthesis pathways, where the synthesis generally starts with the transfer of a sugar-1-phosphate from a uridine diphosphate (UDP)-sugar to an undecaprenyl phosphate (Und-P) molecule in the inner leaflet of the inner membrane (IM) to form an Und-PP-sugar molecule (Whitfield and Trent, 2014). Further work is needed to extend it to other biosynthetic processes involved in bacterial polysaccharide biosynthesis, such as the synthase-dependent pathway (Whitfield and Trent, 2014). Furthermore, the glycosyltransferase OTase has a distinct preference for sugars bearing hexosamines at the reducing end, therefore limiting the pool of polysaccharides that can be used. In addition, only carrier proteins containing the consensus sequence can be glycosylated by PGCT, despite the proper sequence could also be introduced by protein engineering (Ihssen et al., 2015).

Anti-carbohydrate antibodies typically have lower affinity (≥ μM) than anti-protein antibodies (nM) (Astronomo and Burton, 2010), due to an unfavorable entropy contribution during the antibody-antigen complex formation. The recognition of foreign polysaccharide molecules by the immune system relies therefore more on avidity effects, enabled through multivalent interactions, and favored by the repetitive backbone of polysaccharides. In this regard, the design of antigens able to call into action B cells producing high-affinity antibodies is considered a promising perspective, as it has been predicted that high-affinity antibodies could be the future in the treatment of emerging infectious diseases (Marston et al., 2018). The low affinity of protein-glycan interactions and its dependence on multivalent interactions can also be addressed by displaying glycans in a polyvalent fashion. This possibility is particularly interesting for conjugates composed by low molecular weight glycans. Within this framework, nanotechnology has been widely exploited with the aim to improve immune responses for applications related to cancer or infectious diseases (Smith et al., 2013). Nanotechnology approaches have been proposed to improve the efficacy of vaccines by enabling better targeting for DCs, allowing co-delivery of adjuvants/immunogenic carriers or other molecules of interest, and enhancing the stability of antigens (Koshy and Mooney, 2016; Zhang et al., 2019). Typical examples of nanostructures that can be used to more effectively manipulate or deliver immunologically active components are virus-like particles (VLPs, self-assembled structures composed of one or more viral capsid proteins, while synthetic VLS are self-assembled from chemically synthesized components), dendrimers, liposomes, and a variety of nanoparticles (**Figure 2D**). For example, liposomes, due to their biocompatibility and low intrinsic immunogenicity and toxicity, are suitable tools for the co-delivery of carbohydrate antigens and proteins (Said Hassane et al., 2009; Gregory et al., 2013; Li et al., 2016; Hill et al., 2018). Likewise, different kinds of nanoparticles have been recently proposed as potential multivalent delivery systems also for carbohydrate-based vaccines (Smith et al., 2013; Irvine et al., 2015; Irvine and Read, 2020). Metal nanoparticles, and in particular gold nanoparticles (AuNPs) have attracted great attention in this field due to their unique characteristics of biocompatibility and easy production (Compostella et al., 2017). In the last years, AuNPs have been tested as carrier for short synthetic oligosaccharides in animal models (Safari et al., 2012; Vetro et al., 2016; Compostella et al., 2017). In the same category fall outer membrane vesicles (OMVs), where a carbohydrate-based antigen is expressed on the surface (**Figure 2D**). In this regard, the glycoengineering of OMVs for expression of heterologous polysaccharides attached to O-antigen negative lipid A core has been reported (Chen et al., 2016; Gerritzen et al., 2017; Valguarnera and Feldman, 2017; Stevenson et al., 2018). Another approach uses vesicles derived from bacteria genetically engineered to release more OMVs, called GMMA (Generalized Modules for Membrane Antigens) (Gerke et al., 2015). GMMA are further modified to attenuate the toxicity of the lipid A portion (Rossi et al., 2014). GMMA are particularly immunogenic as they allow antigens to be presented in their native context and conformation and because they possess intrinsic adjuvant properties due to the TLRs agonists expressed on their surfaces. GMMA platforms have been used for several O-antigen-based vaccine candidates, including *Shigella sonnei*, which have been tested in Phase 1 and 2 clinical trials showing to be well tolerated and immunogenic (Launay et al., 2017; Obiero et al., 2017). GMMA can also be carriers for heterologous antigens, introduced either by genetic engineering (Micoli and MacLennan, 2020) or by chemical conjugation (Micoli et al., 2020a; Necchi et al., 2021). To sum up, OMVs can be manipulated to express glycans or proteins from a pathogen of interest and their use allows simplified manufacturing processes, lower analytical controls, and overall reduced cost of production (Kis et al., 2019). In recent years, other noteworthy technologies have emerged for the development of novel carbohydrate-based vaccines against infectious diseases. The codelivery of the two components of a conjugate vaccine – sugar and protein – by non-conventional connections has been proposed (**Figure 2E**). To this end, the Protein Capsular Matrix Vaccine (PCMV; Matrivax) technology allows to capture both the sugar antigen and the carrier protein in a crosslinked polymer matrix (Thanawastien et al., 2015). Differently, in the ‘MAPS’ (Multiple Antigen Presenting System) technology, covalent binding is replaced by affinity-based coupling of proteins and biotylinated polysaccharides. The latter methodology was used for a pneumococcal vaccine prototype and has now been extended to *S*. Typhi, *Staphylococcus aureus*, *Klebsiella pneumoniae* and *Pseudomonas aeruginosa (* Zhang et al., 2013).

### Recent Mechanistic Studies on the Immune Response to Glycoconjugate Vaccines

The analysis of the variables that influence the immunogenicity of conjugate vaccines needs to be complemented with in-depth mechanistic studies to better understand the interaction of glycoconjugates with the immune system, to unravel the reasons of the structure-immunological activity relationships of different vaccine prototypes and, ultimately, to develop more effective vaccines. Glycoconjugate vaccines have been victim of their own success, as their tremendous impact on public health has perhaps contributed to their semi-empirical development. In the last years, an increasing number of contributions have shown how a better understanding of the principles governing the interaction of glycoconjugates with the immune system can lead to an improved generation of vaccines. For example, the discovery of T cells that recognize only the carbohydrate portion of the glycoconjugate vaccine, has led to the design of new-generation vaccines enriched in the number of glycan-peptide epitopes that induce more protective antibodies compared to a standard glycoconjugate vaccine (Avci et al., 2011). More recently, it has also been reported that different mechanisms of presentation, based on the structure of the carbohydrate antigen, are operative in responses to glycoconjugate vaccines (Sun et al., 2019). Several glycoconjugates were tested for their ability to induce Tcarb-dependent responses. Almost all conjugate vaccines tested (i.e. *S*. Typhi Vi CPS, Group B Streptococcus type Ib, Hib) induced Tcarb-dependent responses with the exception of group C *N. meningitidis* glycoconjugate. In the latter case, only peptides generated from the carrier protein were critical for helper T cell recognition. Digestion of this acid-sensitive polysaccharide, a linear homopolymer of α(2 → 9)-linked sialic acid, to the size of the monomeric unit resulted in a dominant CD4+ T cell response to peptides in the context of MHCII. This research has highlighted how an understanding of the mechanisms underlying the immune responses to glycoconjugates is crucial in the production of highly protective knowledge-based vaccines. In another recent work, the discovery of a size-dependent factor affecting the relative affinity of antibodies induces by the LPS O Antigen (OAg) of *Francisella Tularensis*, led to the design of a glycoconjugate bearing a genetically-enlarged OAg which provided significantly greater protection than conjugate vaccines produced using smaller OAgs, despite inducing lower IgG titer (Stefanetti et al., 2019). Protective antibodies were found to recognize a length-dependent epitope better expressed on larger sized OAg, which bind with higher affinity to the organism. This observation challenges the paradigm of a direct correlation between the amount of IgG induced by a glycoconjugate and the level of protection conferred, encouraging the development of conjugate vaccines inducing high-affinity antibodies to important pathogens. Interestingly, another recent report challenged instead the use of long polysaccharide, at least for *S.* Typhi conjugate vaccines using the Vi CPS as antigen (Micoli et al., 2020b). It was observed that, while the long-chain-conjugated Vi (165 kDa) induced a response in both wild-type and T cell-deficient mice -suggesting a TI response - short-chain Vi (9.5 to 42.7 kDa) conjugates induced a response in wild-type mice but not in T cell-deficient mice - suggesting a TD response. In addition, long-chain, but not short-chain Vi conjugate induced late apoptosis of Vi-specific B cells in spleen and early depletion of Vi-specific B cells in bone marrow of neonatal mice, resulting in hyporesponsiveness and lack of long-term persistence of Vi-specific IgG in serum and IgG+ antibody-secreting cells in bone marrow. The work suggested that conjugation of long-chain Vi generates an antigen inducing both T-dependent and T-independent responses, while the short-chain Vi conjugate only sustains T-dependent responses. However, it is important to recognize that the two observations mentioned above about the relevance of saccharide size in the context of conjugate vaccine are not inherently conflicting. Indeed, it is known that the impact of polysaccharide size on the immunogenicity of the corresponding conjugate vaccines can be antigen-specific (Jones, 2005; Costantino et al., 2011). The “optimal” size for a conjugate vaccine is likely to be not only antigen-related but also dependent on both the conjugation chemistry and the carrier protein used, as this impacts the final size, configuration and conformation of the glycoconjugate immunogen and how it will be seen and processed by the immune system.

Also, the field of carbohydrate vaccine design is facing huge changes thanks to recent technological and scientific advances. New emerging techniques are helping in the analysis of surface polysaccharide structures (Stojkovic et al., 2017) and to decipher carbohydrate-protein interactions (Ardá and Jiménez-Barbero, 2018), as well as advancement in the field of analytics and immunoassays are assisting the design of glycoconjugate vaccines.

Overall, new advances in the glycomics research are helping to better address the challenges inherent to developing carbohydrate vaccines. These new advances will help to encompass a broader spectrum of diseases, in addition to bacterial infections, including other pathogens – such as viruses, fungi, protozoan parasites, helminths – and, possibly, anti-cancer vaccines.

## Adjuvants for Carbohydrate-Based Vaccines

Adjuvants increase innate immune responses to vaccine antigens and have been used in immunization since the 1930s. Adjuvants are used for several purposes, including: 1) enhancement of vaccine immunogenicity, leading to antigen sparring (i.e. use of a lower dose), reduction of immunizations required for protective immunity, restoration of the response in non- or less-responding individuals; 2) broadening of the response of the immunogen to other antigens; 3) increase in the stability of the formulation (Petrovsky and Aguilar, 2004).

The only adjuvants authorized for conjugate vaccines are aluminum salts (aluminum phosphate or aluminum hydroxide). However, not all the licensed vaccines contain an adjuvant, such as the Food and Drug Administration (FDA) authorized meningococcal conjugate vaccines (**Table 1**). Indeed, the immunological benefit of adding adjuvants to the formulation in preclinical studies is not always replicated in clinical studies and regulators have recently required that the beneficial effect show evidence in clinical settings before authorizing adjuvants’ use. Among the aluminum salts, the most used adjuvant is aluminum phosphate. Introduction of new adjuvants and formulations is difficult due to the stringent safety requirements for vaccines, especially targeting healthy infants. As for polysaccharide vaccines, there are currently no authorized adjuvants, and aluminum salts have been reported to have poor immunological effects (Khandke et al., 2018).

### Aluminum Salts in Glycoconjugate Vaccines

Insoluble aluminum salts were licensed for human use in 1932 and were the only adjuvant on the market for the next 70 years (Di Pasquale et al., 2015). To date, aluminum salts are the most widely used type of adjuvant in human vaccines, mainly due to their beneficial effect on different vaccine formulations, cost effectiveness, and the outstanding safety record in a wide variety of childhood vaccines (Reed et al., 2013). The mechanism of aluminum salts’ interaction with the immune system is not yet fully understood. It has been hypothesized that its adjuvanticity is due to two main causes: 1) a “depot effect” of the antigen at the injection site, with consequent the slow release of the immunogen from local tissues, which prolongs the exposure of immune cells to the antigen, induces the activation of the immune system and also facilitates the uptake of antigen by APCs**;** 2) the release of DAMPs such as DNA (Marichal et al., 2011; McKee et al., 2013), uric acid (Kool et al., 2008), and ATP (Riteau et al., 2012), which collectively result in the recruitment of inflammatory cells into the muscle, with the initial accumulation of neutrophils, followed by monocytes, macrophages and eosinophils (Kool et al., 2008; Riteau et al., 2012). The recruited innate immune cells then take up the antigen and mediate its transport to the draining lymph node, where they orchestrate the adaptive immune responses (O’Hagan et al., 2020).

The physical properties of aluminum salts and their interaction with glycoconjugates are important parameters that influence the immunogenicity of vaccines (Khandke et al., 2018). The glycoconjugate binds to the aluminum salts mainly through the protein moiety. The formulation of the antigen with aluminum adjuvants should be performed with care, as a too strong binding can compromise both the antigen exposure to the immune system and the stability of the vaccine. Among the factors influencing this process, it is worth mentioning: the formulation procedure, the methodology used for producing the adjuvant (particularly true for aluminum phosphate), the presence of excipients, the amount of aluminum, the presence of multiple conjugates with varied levels of saccharide to protein ratios, the overall surface charge of the antigen, tertiary structures and steric hindrance. The final vaccine suspension may switch between flocculated and deflocculated states, depending on the formulation conditions and the associated electrostatic interactions of the adjuvant particles. The sedimentation behavior of aluminum phosphate suspensions has been correlated to formulation parameters including pH, ionic strength, and the presence of model antigens (Muthurania et al., 2015). The particles of aluminum with the immunogen can be reduced in size, resulting in a variation in the electrostatic interactions or flocculation behavior, morphology, and the surface properties (charge, viscosity, surface tension, etc.) which may lead to a change of the visual characteristics of the product and to a different resuspension profile (Hem and White, 1995; Maa and Hsu, 1996; Lindblad, 2004; Rowe et al., 2006; Muthurania et al., 2015; Kolade et al., 2015). It is important to measure all the physical parameters during the formulation process to determine optimal mixing conditions and the impact of shear on the aluminum particles. Many biophysical analyses can be performed to understand the physical characteristics of the aluminum-containing vaccines, including isothermal titration calorimetry (ITC), front-face fluorescence spectroscopy, differential scanning calorimetry (DSC) and Fourier-transform infrared spectroscopy (FTIR) (Khandke et al., 2018).

### New Adjuvants for Carbohydrate-Based Vaccines in Trial

While the use of adjuvants initially relied mainly on empirical evidence, over the past decade their design became increasingly customized to achieve an optimal immune response, with the inclusion of new formulations in licensed products (Di Pasquale et al., 2015; O’Hagan et al., 2020). This is mainly due to the development of systems vaccinology approaches which have simplified the identification of the immunomodulatory mechanism of action. Several new vaccines that contain novel adjuvants, including AS01 (Shingrix, Shingles and RTS,S, Malaria), AS03 (Pandemrix, pandemic Flu), AS04 (Cervarix, HPV) MF59 (Fluad, seasonal Flu) and immunostimulatory oligonucleotides (ISS 1018 in Heplisav-B, HBV) have recently been licensed, and other adjuvants are also going through the later stages of clinical development (O’Hagan et al., 2020), also for COVID-19 vaccines (Chung et al., 2021; COVID-19 Vaccine Tracker and Landscape, 2021). The gradual replacement of inactivated whole cell and live attenuated vaccines with subunit vaccines, such as polysaccharide and glycoconjugate ones, which only contain a purified component of the pathogen, has improved safety profile, production feasibility and analytical characterization of the immunogen. However, purity and precision came at a cost, with the need to incorporate adjuvants to elicit a stronger and/or longer protective immune response.

Unfortunately, with regard to conjugate vaccines, despite promising evidence in animal models, experience in humans have shown that it is difficult to evaluate the increase in immune response using an adjuvant, in particular in primed or pre-exposed adolescents and adults (Paoletti et al., 2001; Søgaard, 2011; Levy et al., 2015; Leroux-Roels et al., 2016). The use of other adjuvants in place of aluminum salts has also not yet been successful. Indeed, the documented human clinical trials conducted so far by using different adjuvants, such as QS 21, MF59, Monophosphoryl lipid A (MPLA) and synthetic oligonucleotides CpG, have not been effective enough to reach the market (Vernacchio et al., 2002; Søgaard et al., 2010; Søgaard, 2011; Garçon and Di Pasquale, 2017). Most of the attention on the use of new adjuvant formulations has focused on pneumococcal conjugate vaccines, where new adjuvants are desirable to boost immune responses, especially for high-risk individuals. While the results were mostly unsuccessful, some positive indications emerged. In a double-blind placebo-controlled clinical trial, randomized HIV-positive patients received two doses of PCV-7 (Prevnar) at 0 and 3 months and one dose of PPV-23 at 9 months. Experimental patients received 1 mg of the TLR9 agonist oligonucleotides CPG 7909 added to each of their 3 vaccine doses; while the control patients had instead phosphate-buffered saline added. The addition of CPG 7909 to PCV-7 doubled the proportion of subjects achieving a high vaccine-specific IgG antibody response at 9 months (pre-PPV-23 immunization). The adjuvant also stimulated a longer lasting antibody response to a greater extent than administration of PCV-7 alone. Furthermore, the final boosting with PPV-23 resulted in increased production of antibodies with higher opsonophagocytic activity in the experimental group than in the control group. However, the use of CPG 7909 to PPV-23 did not enhance the antibody response to non-PCV-7 serotypes and mild systemic and injection-site reactions to PCV-7 were more common in the experimental group. In another study, the use of the TLR9 agonist CPG 7909 has also shown to induce cellular memory to pneumococcal conjugate vaccines (Offersen et al., 2012). HIV-patients were immunized twice with pneumococcal conjugate vaccine (PCV-7) with or without CPG 7909. After vaccination, peripheral blood mononuclear cells were stimulated with pneumococcal polysaccharides and cytokine production measured. The study demonstrated that the CPG 7909 adjuvant increased cytokine responses for IL-1β, IL-2R, IL-6, IFN-γ and MIP-β, which did not correlate with IgG antibody responses. These findings suggests that CPG 7909 induces T cell responses to pneumococcal polysaccharides in HIV-patients, independently of the humoral response. The activation of T helper type 1 (Th1) phenotype may be quite relevant given that newborn and infants exhibit an impaired Th1 response, which could explain their lower response to vaccines and increased vulnerability to infections (Levy, 2007; Kollmann et al., 2012). Similar results were found in a clinical study evaluating the safety and adjuvant properties of MPLA, a detoxified version of *Salmonella minnesota* LPS which exerts its immunomodulatory function primarily by activating the TLR4 (Vernacchio et al., 2002). Toddlers were immunized with two doses of nine-valent pneumococcal–CRM197 conjugate vaccine (PCV9) and cell-mediated responses, examined after the boost, demonstrated a dose-dependent effect of MPLA on Th1 responses to the carrier protein and suggested an effect on T-helper cell type 2 (Th2) responses. However, MPLA did not significantly enhance the concentration of IgG pneumococcal capsular polysaccharide antibody as compared to the aluminum salt control group.

Notwithstanding the difference that may arise when comparing the use of adjuvants in preclinical vs clinical setting, it is worth mentioning some interesting novel adjuvants developed in recent years for carbohydrate-based vaccines with promising results in animal models. A recent work examined the use of Alum-TLR7, based on a TLR7 agonist (SMIP7.10) adsorbed to aluminium hydroxide, for glycoconjugate antigens of different *N. meningitidis* strains (Wu et al., 2014; Buonsanti et al., 2016). The use of Alum-TLR7 outperformed aluminium hydroxide alone for both a monovalent and a tetravalent anti-meningococcal vaccine. Compared to aluminium hydroxide, Alum-TLR7 increased immunogenicity of MenC-CRM197 already after one immunization, induced higher titer of functional antibodies, and shifted the response toward a Th1 phenotype. Another interesting new approach conceives the use of self-adjuvanting glycoconjugate vaccines, by conjugating the antigen with strong adjuvants to overcome the lack of immune stimulation (Li and Guo, 2018). Since the antigen and the adjuvant are covalently linked, the immune cells uptake them simultaneously and thus the activity of the adjuvant can be tailored specifically to the cells of interest. Importantly, self-adjuvanting vaccines have been shown, at least in some context, not to require co-administration of additional adjuvants nor conjugation to carrier proteins. While most applications of self-adjuvanting conjugates regard antitumor vaccines, there are some interesting examples concerning vaccines to prevent infectious diseases. In this regard, the *S. pneumoniae* type 14 tetrasaccharide conjugated to the natural killer T cell adjuvant αGalCer was able to produce an immune response superior to a clinically used vaccine (Prevnar) (Deng et al., 2014). The mechanism of immunomodulation was proposed in a previous report, and involve interaction of B cells with a specific subset of innate-like T cells, namely natural killer T (NKT) cells (Bai et al., 2013). Using liposomal nanoparticles displaying synthetic lipid and polysaccharide antigens to elicit pure and direct NKT–B-cell interactions *in vivo*, the authors observed intense and prolonged antibody responses with isotype switch, affinity maturation, and long-lasting B-cell memory, despite modest or absent NKT follicular helper differentiation. Furthermore, they demonstrated a requirement for a two-step process involving first cognate interactions with DCs, for NKT cell activation, and then with B cells, for induction of isotype switch and memory. Thus, NKT help to B cells represents both a major arm of antimicrobial defense and a promising target for B-cell vaccines. More recently, the synthesis of glycoconjugates for *N. meningitidis* serogroup C prepared by using synthetic α-(2 → 9)-linked di-, tri-, tetra-, and pentasialic acids conjugated to MPLA has been reported (Liao et al., 2016). MPLA glycoconjugates were administered to mice as liposomal formulations and elicited robust immune responses comparable to those induced by the traditional glycoconjugates with adjuvant. Another study extended the use of MPLA-based self-adjuvanting conjugates to antituberculosis vaccines. It was found that the tetrasaccharide of mycobacterial LAM conjugated to the primary position of the glucosamine residue of MPLA induced a robust IgG response in mice. Interestingly, the structure of the linker and the conjugation site of the carbohydrate antigen epitope on MLPA influenced the immunogenicity of the construct (Wang et al., 2017). Novel delivery systems, such as liposomal antigen delivery, DCs, and OMVs, can also have an adjuvant effect on the immunogenicity. One recent example is the use of synthetic liposomes displaying at their surface both the antigen and the adjuvant. The authors reported the design of chemically defined diepitope constructs consisting of liposomes displaying at their surface synthetic oligosaccharides mimicking the O-antigen of the *Shigella flexneri* 2a LPS (B cell epitope) and influenza hemagglutinin peptide HA 307–319 (T cell epitope). The two epitopes were coupled to the lipopeptide Pam3CAG (TLR2 ligand) attached in preformed vesicles. In mice, these synthetic liposomes induced antibody responses against the native LPS.

Another interesting strategy for improving vaccine responses is the combination of different adjuvants activating different receptors of the immune system. In a recent publication, the STING agonist 3’3’-cyclic GMP-AMP (cGAMP) and the soluble TLR7/8 agonist resiquimod (R848) were co-encapsulated within acetylated dextran (Ace-DEX) microparticles (MPs) *via* electrospray (Collier et al., 2018). Using the ovalbumin as antigen model, it was observed in mice that the coencapsulated adjuvant system induced antigen-specific cellular immunity and a balanced Th1/Th2 humoral response greater than both cGAMP Ace-DEX MPs alone and PAMPs delivered in separate MPs. In another study, a protein-based nanovaccine encapsulating an adjuvant combination of R848 and muramyl dipeptide (MDP) was shown to trigger strong additive dendritic cell stimulation and strong antigen-specific CD4+ and CD8+ T cell proliferation (Paßlick et al., 2018). However, in the presence of carbohydrate antigens, the combination of different adjuvants aimed at activating multiple PRRs can also lead to negative outcomes, as it has been observed in the context of MPLA-based self-adjuvanting vaccines (Wang et al., 2012; Liao et al., 2016). Mechanistic investigations are therefore needed to better understand how the combination of multiple adjuvants can improve the design of carbohydrate-based vaccines. Finally, it is important to emphasize the importance of clinical studies in humans to directly compare the effectiveness of glycoconjugate vaccine with new adjuvant strategies.

## Carbohydrates as Vaccine Adjuvants

Carbohydrates and carbohydrate-containing molecules exert potent immunomodulatory effects on the innate immune system and have therefore shown adjuvant activity in pre-clinical and clinical studies (Pedersen et al., 2018; Brown et al., 2018; Lang and Huang, 2020; Garcia-Vello et al., 2020; Pifferi et al., 2021). Carbohydrates interact with the innate immune system in a variety of ways, and often their mechanism of action is not completely understood. Nevertheless, at least some of these molecules can activate innate immune receptors, such as PRRs, or in some cases an invariant TCR a chain expressed by NKT cells. In both instances, activation of innate immune cells leads to production of cytokines, chemokines and expression of co-stimulatory molecules that are important for initiating the adaptive immune response upon vaccination. Alternatively, PRR-mediated recognition of carbohydrates linked to antigens can modulate antigen kinetics and targeting to specific immune compartments (Wilson et al., 2019; Tokatlian et al., 2019). Finally, carbohydrates have also been used as delivery system to encapsulate antigens and promote their uptake by APCs. Here we will outline some carbohydrates that hold promise as vaccine adjuvants focusing on established concepts and unexplored areas of their mechanism of action.

### Glucans

**Figure 3:**
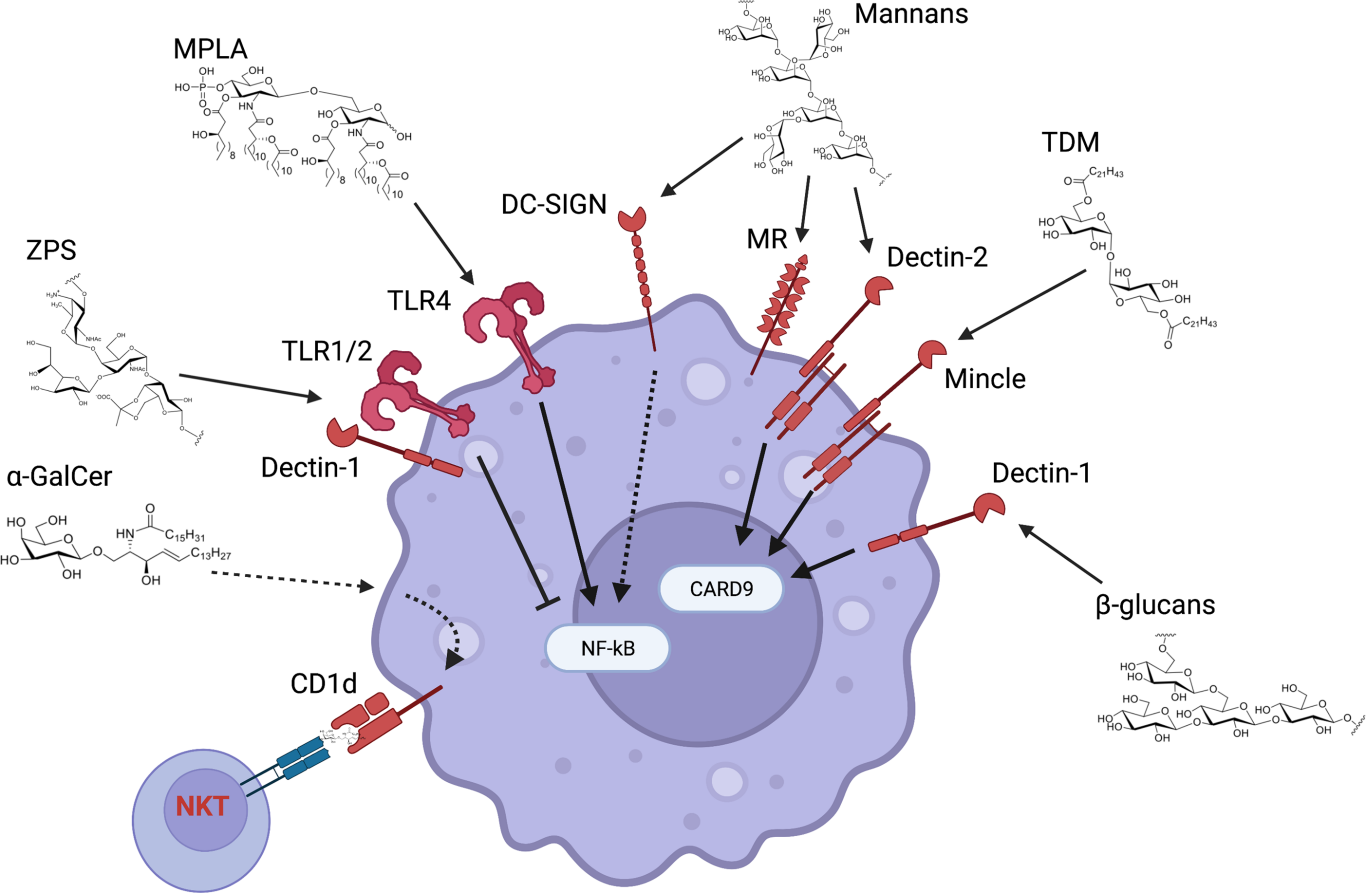
Innate receptors and pathways activated by carbohydrates as vaccine adjuvants. Carbohydrates that have been tested as vaccine adjuvants can activate a multitude of receptors and downstream pathways. β-glucans and mannans respectively bind to Dectin-1 and -2 and lead to CARD9 activation (even though CARD9-independent pathways have also been described). CARD9 plays a key role in NF-κB activation and therefore in the induction of pro-inflammatory genes. A similar pathway is activated by Mincle agonists such as trehalose 6,6’-dimycolate (TDM). Mannans also bind to mannose receptor (MR) and DC-SIGN. MR has mainly been involved in endocytosis and it is unclear whether it activates downstream pathways, while DC-SIGN enhances NF-κB activation upon concurrent TLR stimulation. Monophosphoryl lipid A (MPLA) activates the TLR4 receptor complex mainly through its lipid chains, with the sugar backbone providing a scaffold for the lipid chains. Zwitterionic polysaccharides such as PSA-1 activate both TLR1/2 and Dectin-1, leading to reduction of NF-κB pro-inflammatory activity. Finally, sphingolipids such as α-GalCer are presented to and activate NKT cells through CD1d but can also directly activate innate immune cells in a CD1d-dependent manner.

Glucans are polysaccharides of D-glucose joined by α- and/or β-linkages. There is an important diversity in their molecular weight and configuration depending on the original source. β-Glucans are β-1,3-linked glucose polymers with β-1,6 branches. β-Glucans can be isolated from several sources including fungal cell wall, bacteria, seaweed and cereal. β-glucans bind the CLR Dectin-1 (**Figure 3**), although some preparations might also activate TLRs. This receptor is expressed by many myeloid cell subsets and therefore β-glucans have been evaluated as vaccine adjuvants, delivery systems or in some cases as antigens for anti-fungal vaccines (Garcia-Vello et al., 2020; Lang and Huang, 2020; Pifferi et al., 2021). Of note, β-glucans can be Dectin-1 agonist or antagonist based on their physical properties and structure, with soluble and particulate β-glucans respectively inhibiting or activating Dectin-1 signaling (Goodridge et al., 2011). The current model of Dectin-1 activation states that β-glucans in a particulate form primes Th1, Th17, and cytotoxic T lymphocyte responses. The β-glucan-Dectin-1 binding activates downstream signaling by inducing clustering of Dectin-1, displacement of regulatory phosphatases CD45 and CD148, and SRC kinase-dependent phosphorylation of the intracellular immunoreceptor tyrosine-based activation motif (ITAM)-like motif of Dectin-1 (Borriello et al., 2020). These events lead to the activation of the kinase SYK which in turn activates CARD9, a key signaling molecule that promotes nuclear translocation of the canonical NF-κB transcription factor subunits p50, p65, and c-REL and induction of proinflammatory genes. SYK also activates the kinase NIK and the noncanonical NF-κB subunits p52 and RELB in a CARD9-independent manner. In addition, SYK-independent activation of the kinase Raf-1 modulates the activity of NF-κB subunits for example by inducing the formation of p65-RELB dimers. Although the functional consequences of these events in the context of Dectin-1 signaling are not completely understood, there is evidence suggesting an important role for Raf-1- and NIK-dependent modulation of NF-κB subunit activity in eliciting a Th1 response through the induction of the proinflammatory cytokines IL-12p40 and IL-1β. Mitogen-activated protein kinases (MAPKs) are also activated downstream of Dectin-1 in a SYK- dependent manner. In particular, the kinase ERK cooperates with NF-κB to modulate the proinflammatory response upon Dectin-1 activation. Dectin-1 signaling also activates the transcription factors IRF1, IRF5, and NFAT. IRF5 is activated in a CARD9-dependent manner and induces IFN-β. NFAT is activated in a PLCγ2/calcium/calcineurin-dependent manner and regulates the expression of a subset of genes such as IL-2 that plays a critical role in NK- and T-cell activation. Overall, Dectin-1 activates several signaling pathways leading to the expression of multiple pro-inflammatory programs that impact T cell differentiation. More work is required to dissect the contribution of these pathways to β-glucans adjuvanticity. It is also worth noting that some soluble β-glucans still retain immunomodulatory function. Careful examination of several preparations of the soluble β-glucan laminarin revealed that some of them are agonists while some others are antagonists, and their activities were not correlated to Dectin-1 binding affinity (Smith et al., 2018). Intravenous administration of soluble β-glucans isolated from the yeast *Saccharomyces cerevisiae* leads to its uptake by macrophage and cleavage of a 25 kDa fragment that binds to complement receptor 3 (CR3) on neutrophils, priming them for targeted killing of tumor cells (Li et al., 2006). Altogether, more structure/activity relationship studies are required to fully understand the biology of β**-**glucans/Dectin-1 interaction. It will also be key to investigate the immunomodulatory potential of particulate and soluble β**-**glucans in different experimental models (e.g., *in vitro* vs *in vivo*) since their activity can be context-dependent.

α-glucans employed in vaccine research are mainly represented by dextran and its modified forms (e.g., acetylated dextran). Dextran has been used as carrier for antigens and/or adjuvants, increasing their uptake by myeloid cells which leads to enhanced antigen presentation to T cells, pro-inflammatory activation, and therefore vaccine efficacy (Lang and Huang, 2020; Garcia-Vello et al., 2020; Pifferi et al., 2021). There is evidence that myeloid cell uptake of dextran is mediated by CLRs, such as mannose receptor (MR) and DC-SIGN (Pustylnikov et al., 2014). However, it is still unclear whether dextran binding to these receptors leads to innate immune cell activation and adjuvant activity.

### Mannans

Mannans are polysaccharides of D-mannose and represent an important component of the fungal cell wall where it mainly presents a backbone of α-(1→6) linked mannose units with α-(1→2)– and α-(1→3)– linked side chains and can be also linked to proteins. Mannans in native, oxidated or reduced form have been tested as adjuvants (either in combination with or conjugated to protein antigens) or as antigens for antifungal vaccines (Lang and Huang, 2020; Garcia-Vello et al., 2020; Pifferi et al., 2021). Mannans are recognized by several CLRs including MR, DC-SIGN and Dectin-2 (Borriello et al., 2020) (**Figure 3**). In particular, MR and DC-SIGN recognize terminal mannans while Dectin-2 binds to high-mannose structures (Feinberg et al., 2007; Feinberg et al., 2017; Feinberg et al., 2021). These receptors also trigger distinct signaling pathways and cellular responses. MR mediates endocytosis and phagocytosis upon phosphorylation of a tyrosine residues in its cytoplasmic tail, but it is still unclear to what extent it activates intracellular signaling pathways. There is evidence that human MR lacks activating ITAMs capable of transducing the signal but is associated with the co-receptor Fc receptor common gamma chain (FcRγ) which contains ITAMs and therefore leads to SYK activation upon *in vitro* macrophage infection with *Mycobacterium tuberculosis (* Rajaram et al., 2017). In addition, a synthetic peptide that induces MR endocytosis and downstream signaling modulates macrophage viability and induces a pro-inflammatory activation program (e.g., TNF, IL-12, IL-1b) (Jaynes et al., 2020). *In vivo* this resulted into enhanced anti-tumor immunity and improved survival in mouse models of cancer and lung fibrosis, respectively (Jaynes et al., 2020; Ghebremedhin et al., 2020). Thus, a better understanding of signaling events downstream of MR might have important implications for a range of diseases as well as for designing effective mannan-based adjuvants.

DC-SIGN does not signal through SYK but it is instead constitutively associated with a signalosome composed of the scaffolding proteins LSP1, KSR1, CNK and Raf-1 (Gringhuis et al., 2009). Binding to mannose-containing molecules leads to Raf-1 activation which in turn increases NF-κB p65 phosphorylation and acetylation upon concurrent TLR activation. These events lead to enhanced DNA binding of nuclear p65 and increased expression of IL-10, IL-6 and IL-12 (Gringhuis et al., 2007). While this has important implication for pathogen recognition and subsequent innate immune activation, it is still unclear whether mannan-based adjuvants will activate these pathways in the absence of concurrent TLR activation. In addition, this model is mainly based on *in vitro* data and therefore more work needs to be done to validate its relevance *in vivo*.

Dectin-2 has a short cytoplasmic tail and therefore associates with FcRγ to initiate SYK-dependent signaling (Sato et al., 2006; Robinson et al., 2009). It also interacts with Dectin-3 to form heterodimers endowed with higher affinity for mannans (Zhu et al., 2013). The signaling cascade downstream of Dectin-2 is thought to be comparable to the one activated by Dectin-1, even though an in-depth analysis of Dectin-2 signaling would be required to address this point (Borriello et al., 2020). In addition, it will be important to assess whether Dectin-2 activation follows the same rules as Dectin-1 activation, namely particulate ligand-induced receptor clustering and signaling.

Overall, combined activation of MR, DC-SIGN and Dectin-2 is likely to mediate adjuvant activity of mannans. It will be important to assess the relative contribution of each receptor to better define the mechanism of action of mannans. Of note, D-mannose monosaccharides or oligosaccharides conjugated with antigens also increases antigen immunogenicity. Conjugates composed of an antigen, mannose and TLR7 ligand are more effectively taken up by DCs and elicit a stronger pro-inflammatory response than conjugates without mannose. This results into effective induction of antigen-specific cellular and humoral responses upon *in vivo* immunization (Wilson et al., 2019). VLPs decorated with mannose monomers are endocytosed by DCs in a DC-SIGN-dependent manner, induce the expression of pro-inflammatory genes in a LSP1- and Raf-1-dependent manner, and elicit antigen-specific Th1 polarization upon *in vivo* immunization (Jarvis et al., 2019; Alam et al., 2021). Finally, conjugation of protein nanoparticles with trimannose moieties is sufficient for promoting their localization to the follicular dendritic cell network of B cell follicles and enhance their immunogenicity, likely through activation of mannose-binding lectin and complement (Tokatlian et al., 2019). Altogether, these studies highlight the promising role of D-mannose and its polymers in enhancing antigen immunogenicity as well as the need for additional mechanistic studies to harness their potential.

### Zwitterionic Polysaccharides

ZPSs are a unique class of polysaccharides that are capable of eliciting a T-cell response after being processed by antigen presenting cells and presented on MHCII molecules. Most of the studies deciphering the mechanism of action of bacterial ZPS immunomodulation involve the polysaccharide A-1 (PSA-1) of the commensal *Bacteroides fragilis*. Upon recognition of PSA-1 by APCs, two main processes take place – polysaccharide recognition by cell surface receptors, and uptake, digestion and presentation by MHCII molecule (Kalka-Moll et al., 2002; Cobb et al., 2004; Wang et al., 2006; Erturk-Hasdemir et al., 2019). Importantly, it was recently demonstrated that a small amount of covalently bound lipid in PSA-1 is required for the initiation of the triggered immune responses (Erturk-Hasdemir et al., 2019). PSA-1 is recognized simultaneously by Toll-like receptor (TLR) 2/1 heterodimer and Dectin-1 on plasmacytoid DC (pDCs) which leads to the induction of the PI3K pathway. The activation of the PI3K pathway leads to the inactivation of the transcription factor GSK3β by a key kinase, Akt. In this way the PI3K pathway suppresses NF-κB–mediated transcription of pro-inflammatory cytokines and promote CREB/CBP-dependent anti-inflammatory gene expression (**Figure 3**). Consequently, pDCs instruct CD4+ T cells to produce anti-inflammatory cytokine IL-10 (Erturk-Hasdemir et al., 2019). Another CLR, DC-SIGN, was reported to respond to PSA in human cells (Bloem et al., 2013), but the mice homolog SIGNR3 did not induce IL 10-producing T cells (Erturk-Hasdemir et al., 2019).

With regard to applications as vaccine adjuvants, a ZPS, obtained through chemical introduction of positive charges into the anionic polysaccharides of Group B Streptococcus, has shown the ability to increase the antibody titer towards unconjugated TT immunization by activating TLR2-expressing APCs leading to better T-cell priming and higher antibody titers (Gallorini et al., 2009). In addition, when conjugated to a carrier protein, ZPS-glycoconjugates showed to induce higher T-cell and antibody responses to both the polysaccharide and the protein components, compared to a standard glycoconjugates made with the native polysaccharide form. The increased immunogenicity of ZPS-conjugates correlates with their ability to activate DCs. The introduction of zwitterionic motifs into the CPS from Group B Streptococcus leading to the activation of mouse and human APCs through a TLR2- dependent mechanism was previously demonstrated (Gallorini et al., 2007).

### Monophosphoryl Lipid A

MPLA is a TLR4 agonist and chemically derived from *S. minnesota* LPS, an important outer membrane component of Gram-negative bacteria and potent innate immune activator (Cochet and Peri, 2017) (**Figure 3**). Treatment of LPS with mild acidic conditions leads to cleavage of lipid A from the oligosaccharide core and hydrolysis of the 1-phosphate group. Therefore, MPLA is composed of a phosphorylated disaccharide that provides a scaffold for fatty acid chains. MPLA is a key component of adjuvants in licensed vaccines, such as adjuvant system (AS) 01b (a liposomal formulation of MPLA and the saponin QS-21) and AS04 (MPLA formulated with aluminum hydroxide) (O’Hagan et al., 2020).

MPLA induces the dimerization of TLR4 with its co-receptor MD-2 which then leads to the adaptor proteins TIRAP and MyD88, and downstream activation of NF-κB and MAPK signaling pathways. In addition, the TLR4-MD-2 complex can be internalized and recruit the adaptor proteins TRAM and TRIF, leading to activation of IRF3 and expression of interferons and interferon-stimulated genes (Cochet and Peri, 2017). It has been suggested that MPLA has a bias toward TRIF-dependent signaling which is required for *in vivo* adjuvanticity and might explain MPLA reduced toxicity compared to LPS (Mata-Haro et al., 2007). Nevertheless, a synthetic TLR4 agonist with structural similarity to lipid A and more pronounced *in vitro* TRIF bias compared to MPLA does not result in increased *in vivo* adjuvanticity (Richard et al., 2020). Therefore, more work is required to understand the signaling events triggered by MPLA and how they relate to vaccine adjuvanticity.

Finally, it is worth noting that while MPLA disaccharide is an important scaffold for the phosphate and acyl group, it is not strictly required for eliciting TLR4 signaling. Structural analogues of MPLA in which the sugar backbone is substituted by other chemical groups that can provide scaffold function retain immunological properties (Romerio and Peri, 2020).

### Trehalose Glycolipids

Trehalose 6,6’-dimycolate (TDM) is a glycolipid component of *Mycobacterium tuberculosis* cell wall and potent agonist of the CLR Mincle which binds both the sugar portion and the hydrocarbon tail of the glycolipid (Khan et al., 2012) (**Figure 3**). Mincle couples with its co-receptor FcRγ, leading to activation of the SYK/CARD9 signaling pathway and production of pro-inflammatory molecules. The marked pro-inflammatory profile of TDM has hampered its clinical use. However, its synthetic analogue trehalose-6,6′- dibehenate (TDB) has lower toxicity and has showed promising vaccine adjuvant activity as liposomal formulation in pre-clinical and clinical studies. Likewise, brartemicin analogues containing long-chain lipids exhibit potent agonist activity toward Mincle that translates into greater *in vivo* adjuvant activity than TDB.

### α-Galactosylceramide

α-Galactosylceramide (α-GalCer) is a synthetic glycolipid belonging to the class of sphingolipids, a group of lipids characterized by a long-chain amino alcohol sphingoid backbone with an amide-bound fatty acyl chain. α-GalCer contains a galactose head group α-linked to a sphingosine chain (18 carbons) which is further linked to the fatty acyl chain (26 carbons). α-GalCer binds to the non-polymorphic MHCI-like molecule CD1d on myeloid cells through the alkyl chains and is then presented to a subset of lymphocytes referred to as NKT cells since they express both NK and T cell markers as well as an invariant TCR-α chain (**Figure 3**). Activation of NKT cells leads to the rapid production of high levels of type 1 (e.g., IFNγ) and type 2 cytokines (e.g., IL-4). Therefore, α-GalCer has attracted interest as potential adjuvant (Ko et al., 2005). At the same time, the simultaneous induction of type 1 and type 2 cytokines has hampered its clinical use. Nevertheless, structure-activity relationship studies have shown that the balance between these two opposing effector functions can be tuned by selective modifications of the sugar and the lipid moiety. It is also worth noting that CD1d cross-linking on innate immune cells elicits pro-inflammatory cytokine production (Yue et al., 2005), and α-GalCer can directly activate myeloid cells and elicit an innate pro-inflammatory response in a CD1d-dependent manner (Loffredo et al., 2014). Additional studies are required to define the multi-faceted immune response elicited by α-GalCer and understand how to fine-tune its activity through selected chemical modifications.

## Conclusion and Perspective

Although the enormous progresses achieved by medicine, infectious diseases are still a major public health concern. Many microorganisms, including bacteria, represent a major cause of morbidity and mortality, and low life expectancy at birth in the developing countries. Indeed, vaccination is considered by the World Health Organization to be the most cost-effective of all potential infectious diseases’ prevention strategies.

Glycoconjugate vaccines are able to prevent devastating infectious diseases such as pneumonia and meningitis. Historically, these vaccines have been developed to overcome the limitations imposed by plain polysaccharide vaccines, and to confer protection and induce boostable responses in children. Over the last 25 years, glycoconjugate vaccines have been introduced to protect against severe infections caused by the encapsulated bacteria Hib, *S. pneumoniae*, *N. meningitidis* and *S.* Typhi. However, despite the success of current vaccination programs, new challenges are on the horizon. Indeed, there is an urgent need for prophylactic intervention against bacterial infection caused by *Clostridium difficile*, enterotoxigenic and extraintestinal pathogenic *E. coli*, or ‘ESKAPE’ pathogens (i.e. *Enterococcus faecium*, *S. aureus*, *K. pneumoniae*, *Acinetobacter baumannii*, *P. aeruginosa*, *Enterobacter species*) *(* Santajit and Indrawattana, 2016), which are important causes of nosocomial infections and that exhibit multidrug resistance and virulence. Moreover, new breakthrough innovations are needed to fight pathogens and diseases beyond bacterial infection, including fungal and viral infections and cancer, also considering the increase in life expectancy and the increased demand for vaccination of the adult and elderly populations (Micoli et al., 2019).

Importantly, the field of carbohydrate vaccine design is undergoing tremendous changes thanks to recent technological and scientific advances. As highlighted in this review, a careful analysis of preclinical and clinical data generated by existing conjugate vaccines and their adjuvants, as well as the design of experiments aimed both at understanding their mechanism of actions and at evaluating the variables important to their immunogenicity, will likely lead to a generation of improved immunogens. In addition, new emerging techniques are helping in the analysis of surface polysaccharide structures (Stojkovic et al., 2017) and to decipher carbohydrate-protein interactions (Ardá and Jiménez-Barbero, 2018), as well as advancement in the field of analytics and immunoassays. Importantly, a recent outlook has shown that most currently recommended vaccination schedules generate only 10-35% of the potential antibody titer, strongly supporting the contention that the full potential of glycoconjugate vaccines has not yet been achieved (Rappuoli, 2018). Higher antibody titers induced by vaccination would also be beneficial to remove more efficiently the bacterial carriage from the upper respiratory tract of the population (Ojal et al., 2017). In addition, the immune response to conjugate vaccines changes depending on age, with naïve infants responding differently from adolescents and adults, an information that could lead to improved age-dependent immunization programs (Rappuoli, 2018).

Starting from an overview of bacterial carbohydrate immunobiology, this review aimed to both pinpoint the state of the art and to reflect on some open scientific questions that could lead to the generation of improved vaccines and adjuvants (**Table 2**). New scientific and technological progress has improved the design of carbohydrate-based vaccines, reduced the time required for their preparation, and simplified their characterization throughout the production process. New technologies for carbohydrate-based vaccines are likely to focus on several areas, including: 1) improved disease protection by enhancing quantity, quality or persistence of antibodies and/or memory responses; 2) new carrier proteins to overcome the issue of reduced immunogenicity against the saccharide antigen due to the ‘carrier epitope suppression’ phenomenon; 3) Structurally-defined conjugates that simplify characterization of the immunogen and reduce batch-to-batch inconsistencies, including semi-synthetic and fully-synthetic vaccines; 4) Artificial (e.g. liposomes, nanoparticles,.) or natural (e.g. OMVs) multivalent technologies with improved targeting of immune cells and allowing co-delivery of key molecules such as adjuvants or carriers; 5) broader protection from diseases by targeting multiple pathogens within a single construct and/or by constructing vaccines inducing cross-reactive antibodies against more than one pathogen; 6) cost-effective vaccines to target underdeveloped and developing countries. Importantly, all technologies presented here (or to be conceived in the future) have different and unique characteristics that could suggest their use depending on the specific context, including the pathogen of interest, the population target, and the adjuvant in use. Reliable animal models are also important for prioritizing vaccines to move to clinical trials, which remain the ultimate test for evaluating the value proposition of new approaches against existing benchmarks. Moreover, as conjugation parameters are strongly interdependent in determining vaccine efficacy, the use of highly structurally defined conjugate vaccines will simplify the investigation of the structure-immunological activity space and the identification of key variables. The analysis of the variables affecting the immunogenicity of conjugate vaccines needs to be complemented with insightful mechanistic studies to better understand the interaction of glycoconjugates with the immune system, to unravel the reasons for the structure-immunological activity relationships and to ultimately develop new immunogens of improved efficacy. In addition, the analysis of clinical samples is essential to better understand the vaccine response in humans, identify mechanism of actions, define correlate of protection, discover new biomarkers, and guide the design of next-generation immunogens. Biobanks of sera and immune cells from vaccinated and infected individuals can also be used to perform both traditional immunological studies and novel system biology investigations, including multi-omics approaches. Furthermore, the use of structural biology methods and the screening with human monoclonal antibodies can allow the identification of protective antigens and epitopes through a process known as “reverse vaccinology 2.0” (Black et al., 2020).

**Table 2:** Open scientific questions on carbohydrate-based vaccines and adjuvants.

Open question	Comment
**How can we improve the design of carbohydrate-based vaccines?**	- Optimization and discovery of technologies mainly focusing on improved immunogenicity, structurally-defined immunogens, multivalent presentations, cost-effective and time-effective platforms - Context-dependent optimization of variables important for the immunogenicity (e.g. antigen, population target, adjuvant) - Investigations on the mechanism of immune responses - Analysis and comparison of the immune response in human samples of both vaccinated and infected individuals by also employing system biology approaches
**How can we enhance the response to carbohydrate-based vaccines using adjuvants (in humans)?**	- Mechanistic investigation on the different role of adjuvants in preclinical vs clinical model to instruct better design and/or delivery of immunomodulators - Explore the use of new adjuvants (e.g MF59 or AS04) in infants’ vaccination - Analyze the specific interaction of carbohydrates with the immune system as their self-adjuvant properties may be related with the difficulty to boost polysaccharide-based antigen with adjuvants and develop novel knowledge-based immunomodulators
**Can carbohydrate and carbohydrate-containing molecules be employed for novel and effective adjuvant formulations?**	- for glycolipid adjuvants, structure-activity relationship studies will be key to define the relative contribution of sugar and lipid moieties to adjuvant activity - it will be important to understand if signaling pathways specifically activated by carbohydrates adjuvants confer any advantages over existing immunomodulators
**Do polysaccharide-based delivery systems exert adjuvant effects?**	- More studies are required to dissect the contribution of direct innate immune cell activation vs efficient myeloid cell uptake of the cargo in enhancing vaccine efficacy
**Can sugar moieties be employed to modulate antigen targeting to myeloid cells and trafficking to lymphoid organs?**	- Additional studies are required to define whether and how protein decoration with sugar moieties can modulate antigen pharmacokinetics and immunogenicity

We have also discussed here the inherent difficulty in modulating the response to carbohydrate-based vaccines using adjuvants in humans (**Table 2**). Aluminum salts, the only licensed adjuvants, have little or no immunological effects with TI-2 antigens, such as polysaccharide vaccines, and may or may not have effect with glycoconjugate vaccines. Also, the use of other adjuvants has not yet been successful (Paoletti et al., 2001; Søgaard, 2011; Levy et al., 2015; Leroux-Roels et al., 2016). Some possible interdependent reasons that may explain the different response observed using adjuvants for carbohydrate-based vaccines in preclinical vs clinical settings are: 1) animals are more “pathogen naïve” while humans may already be primed for the specific pathogen at the time of vaccination, especially adolescents and adults; 2) differences in immune cell compositions, including population, frequencies and signaling pathway and expression of PRRs; 3) age-related immune maturation factors; 4) the need for a different timing for optimal administration of the adjuvant. Another possibility is that adjuvants may play a more decisive role for carbohydrate-based vaccines in the context of immunocompromised subjects who usually responds very poorly to immunization than healthy individuals, as previously reported (Søgaard et al., 2010). As stated above, the age factor in human is likely related to the possibility that they have already been exposed to the pathogen, and the immunomodulatory effect of adjuvants may be different in priming vs boosting settings. Indeed, the use of MF59 or AS04 in infant baboon and mice induces a better immune response than either unadjuvanted conjugates or aluminum salts-adjuvanted conjugates. Although comparable clinical data in infants are not yet available, it is possible that similar findings would occur in humans. However, in adolescents and adults, the use of adjuvants, including MF59 or AS04, do not improve the immune response (Costantino et al., 1992; Leroux-Roels et al., 2016; Rappuoli, 2018). If this effect were also confirmed in humans, it could lead to the use of non-aluminum salts adjuvants in infants to achieve better protection perhaps even with lower vaccine doses. Oil-in-water emulsions such as MF59 have been already used in infants or children for HIV vaccines, during the H1N1 pandemic or for influenza vaccines (Rappuoli, 2018).

In contrast to carbohydrate-based vaccines, the use of novel adjuvants for other types of vaccine, including the subunit protein-based vaccines, has shown significant progress in recent years leading to new products authorized on the market or currently under investigation in clinical trials (O’Hagan et al., 2020). The reason for this different behavior is likely related to the nature of carbohydrate antigens. Polysaccharides are TI-2 antigens characterized by large molecular weight, repeating antigenic epitopes, ability to activate the complement cascade, poor *in vivo* degradability, and inability to stimulate T-dependent responses. B-cell receptor crosslinking through binding of repetitive motifs activates antigen-specific B cells independently of CD4+ helper T cells. Most adjuvants used so far in conjugate vaccines do not directly activate naïve B cells but target T cells, MBCs and APCs (Søgaard, 2011). For example, the aforementioned CPG 7909 activates pDC and mature B cells *via* the intracellular pattern recognition receptor TLR9 (Krieg, 2006) and has been shown to act as a potent Th1 stimulator adjuvant in combination with other vaccines such as the protein-based hepatitis B vaccine (Daubenberger, 2007). The specific interaction of carbohydrates with the immune system underlies their self-adjuvant properties and probably the difficulty of enhancing polysaccharide-based antigen responses with adjuvants and should be explored in greater details with targeted mechanistic studies.

Preclinical and clinical data also support the use of polysaccharides as vaccine adjuvants (Lang and Huang, 2020; Garcia-Vello et al., 2020; Pifferi et al., 2021). Some molecules like MPLA are key components of adjuvant formulations that are part of licensed vaccine (O’Hagan et al., 2020). However, the sugar component functions mainly as scaffold for the acyl chains which is key to trigger TLR4-mediated signaling (Cochet and Peri, 2017; Romerio and Peri, 2020). Several molecules that have shown adjuvant activity in pre-clinical and in some cases clinical studies, such as α-GalCer and TDB, are glycolipids and therefore structure-activity relationship studies will be key to define the relative contribution of sugar and lipid moieties to adjuvant activity (Pifferi et al., 2021). A different situation is presented by β-glucans and mannans that are pure polysaccharides and trigger CLR-dependent signaling through recognition of sugar motifs (Brown et al., 2018; Borriello et al., 2020). An important question that will need to be addressed is whether signaling pathways specifically activated by polysaccharides confer any advantages as vaccine adjuvants. For example, β-glucans, mannans and TDB activate CARD9-dependent pathways that might lead to distinct innate immune profiles and therefore T cell polarization. A unique case is represented by α-GalCer and related sphingolipids that specifically activate NKT cells through CD1d-mediated presentation. More studies are required to define whether activation of these cellular and molecular pathways confers clinical advantages over non-polysaccharide molecules that trigger different PRRs (e.g., TLRs, RLRs). The potential synergism between polysaccharide molecules activating CARD9-dependent pathways and additional molecules triggering receptors that do not signal through CARD9 is also under investigation and hold promises for the identification of potent adjuvant formulations with unique immunomodulatory functions (van Haren et al., 2016).

Several polysaccharides have also been employed as delivery systems by encapsulating antigens and/or adjuvants and therefore increasing myeloid cell uptake (Lang and Huang, 2020; Garcia-Vello et al., 2020; Pifferi et al., 2021). Since this event is key for innate immune cell activation and antigen presentation, polysaccharides can greatly enhance vaccine efficacy. It is worth noting that some of these polysaccharides such as the α-glucan dextran can also activate CLRs (Pustylnikov et al., 2014). There is also evidence that chitosan, a polysaccharide composed of β-(1 → 4)-linked *N*-acetyl-d-glucosamine and d-glucosamine employed as delivery system, exerts a direct adjuvant effect by activating innate immune cells through intracellular release of DNA and triggering of the cGAS/STING pathways (Moran et al., 2018). More studies are required to assess whether the enhance vaccine efficacy observed with polysaccharide delivery systems can also be attribute to direct innate immune cell activation by polysaccharides rather than only more efficient myeloid cell uptake of the cargo.

Decoration of protein antigens with sugars is also an effective strategy to promote antigen immunogenicity. Antigen conjugation with mannose monomers or oligomers can have a broad impact on the innate immune compartment, ranging from enhanced CLR-mediated uptake by and activation of innate immune cells to complement deposition and prolonged permanence in lymph node GCs (Wilson et al., 2019; Tokatlian et al., 2019; Jarvis et al., 2019; Alam et al., 2021). These events shape both the magnitude and the polarization of the antigen-specific immune response. Overall, mono-/oligosaccharide conjugation with protein antigens may lead to targeting of specific innate immune compartments and/or triggering specific signaling pathways and effector responses. While the precise mechanisms are still not completely defined and likely depend on antigen-specific properties as well, this approach might represent a relatively easy way to harness sugars as both innate immune cell activators and delivery systems to improve antigen immunogenicity.

In summary, we have reviewed recent advances in the development of vaccines that use carbohydrates as antigens and/or adjuvants. We have explored the ability of glycans to steer both the innate and adaptive arms of the immune response and reflected on the next-generation of knowledge-driven carbohydrate-based immunogens. We believe that new advances in glycomics will foster a new era of improved interventions against infectious diseases.

## Author Contributions

GS conceived the idea and the workflow of the review. All authors contributed to the writing and discussion

## Funding

The work was supported founded by the National Institute of Health (R01 AI148273 grant).

## Conflict of Interest

FB and IZ are named inventors on invention disclosures and patents involving vaccine adjuvants.

The remaining authors declare that the research was conducted in the absence of any commercial or financial relationships that could be construed as a potential conflict of interest.

## Publisher’s Note

All claims expressed in this article are solely those of the authors and do not necessarily represent those of their affiliated organizations, or those of the publisher, the editors and the reviewers. Any product that may be evaluated in this article, or claim that may be made by its manufacturer, is not guaranteed or endorsed by the publisher.
